# Board Network and CSR Decoupling: Evidence From China

**DOI:** 10.3389/fpsyg.2022.815341

**Published:** 2022-03-11

**Authors:** Weiqi Zhao, Ma Zhong, Xinyi Liao, Chuqi Ye, Deqiang Deng

**Affiliations:** School of Economics and Management, Nanjing Forestry University, Nanjing, China

**Keywords:** decoupling, environmental, social and governance (ESG), CSR washing, symbolic and substantive strategy, emerging market, director social network

## Abstract

This paper investigates the influence of board network centrality on corporate social responsibility (CSR) decoupling. CSR decoupling refers to the gap between corporate internal and external actions in CSR practices. Specifically, we measure CSR decoupling as the difference between corporate social disclosure (CSD) and corporate social performance (CSP). This paper uses a sample of Chinese A-share listed firms during 2009–2018, takes the technical dimension score (T-score) and content dimension score (C-score) of RKS ratings as proxies of CSD and CSP, and obtains CSR decoupling as the difference between CSD and CSP. Our results show that (1) board network centrality is positively related to over-decoupling in the pre-adoption period (2009–2014) of the new environmental law but negatively related to over-decoupling in the post-adoption period (2015–2018) and (2) centrality is not related to under-decoupling in the pre-adoption period but a significantly positive related in the post-adoption period. Our finding reveals a complex role of the board network in CSR practices in China.

## Introduction

Previous studies based on network theory find that social networks built by top managers, e.g., CEOs and directors, affect not only corporate financing, investment, and other traditional business practices (Chuluun et al., 2014; Feng et al., 2019) but also corporate social responsibility (CSR) practices. For example, Harjoto and Wang (2020), Lai et al. (2020), and Nandy et al. (2020) find that boards with higher network centrality can bring social capital to the firm and stronger advantages in information access and exchange, which helps firms to improve corporate social performance (CSP). However, these studies implicitly assume that the firm discloses its CSP truthfully and no misalignment between its CSP and corporate social disclosure (CSD). In the real world, the existence of information asymmetry, moral hazard and so on leads to a misalignment between CSD and actual CSP, that is, CSR decoupling (García-Sánchez et al., 2020; Sánchez et al., 2021; Shahab et al., 2021). Specifically, some firms adopt symbolic management in their CSR practices and tend to disguise and exaggerate their actual CSP levels by making excessive and selective CSD (Walker and Wan, 2012; Mahoney et al., 2013; Yu et al., 2020). On the other hand, high CSD may be a stimulus for firms to face higher social expectations and legal pressure, and they may have incentives to reduce the CSD that matches their actual CSP (Carlos and Lewis, 2018). Thus, a research question is generated: do firms use their advantages of board networks to increase or decrease the misalignment between CSD and CSP, specifically, the positive or negative gap between CSR disclosure and performance? For example, firms may take advantage of social networks to reinforce the application of symbolic strategies, thereby widening the positive gap, or they may take advantage of social networks to mitigate social expectations and legal pressure, and make more CSD, thereby reducing the negative gap.

As the most important developing market in the world, the Chinese economy has begun to change from barbaric growth to sustainable growth in recent years. Since the implementation of the mandatory CSR reporting policy in 2009, the Chinese CSR system has achieved great development (Yin and Zhang, 2012; Shen et al., 2020), and socially responsible investors (SRIs) have sprung up (SynTao., 2019). However, some deficiencies still exist in the CSR regulatory systems, such as weakly related litigation and public opinion supervision systems, a lack of detailed reporting guidelines, information assurance, influential CSR ratings and executable regulatory policies (Situ and Tilt, 2018; Yin and Quazi, 2018; Wu and Pupovac, 2019). Therefore, Chinese firms still have enormous discretion in the breadth, depth, and quality of their CSD. Situ et al. (2018) shows that the environmental policies of the Chinese government can only affect whether firms disclose CSR information, but the impact on the level of disclosure is extremely limited. This “excessive freedom” causes a terrible problem of the decoupling between CSP and CSD, which troubles market regulators and participants (Zhang and Chen, 2019).

Based on Chinese A-share firms listed on the Shanghai and Shenzhen stock exchanges for the period of 2009–2018, we provide evidence for the relationship between board network centrality and CSR decoupling. We use the mean value of four network centrality indicators namely, degree centrality, closeness centrality, betweenness centrality, and eigenvector centrality, after sorting them into 10 quantiles as proxies of board network centrality. CSR decoupling is measured as the difference between the CSD and CSP. We use the standardized technical dimension score (T-score) and content dimension score (C-score) provided by Rankins Ratings (RKS) as proxies for CSD and CSP. Over-decoupling and under-decoupling indicate that a firm has disclosed too much or less in CSD compared with the actual CSP. Our analysis suggests that board network centrality has a significantly positive (negative) influence on over-decoupling in the pre-adoption (post-adoption) period of the new environmental law. Meanwhile, board centrality is not related to under-decoupling in the pre-adoption period but significantly positive related in the post-adoption period.

Our study makes three main contributions. First, we contribute to the CSR literature based on network theory. Previous studies have suggested that there is a positive relationship between board networks and CSP (Harjoto and Wang, 2020; Lai et al., 2020; Nandy et al., 2020), but little is known about the impact of board networks on CSR decoupling. We argue that board network centrality plays a complex role in CSR practices of China.

Second, this paper examines the role of foreign investors in CSR decoupling in China and enriches the understanding of corporate governance mechanisms in emerging markets. The existing literature has confirmed the impact of foreign investors on the CSR practices of Chinese firms (McGuinness et al., 2017; Li et al., 2021). Our evidence shows that foreign investors play a vague role in the relationship between board centrality and CSR decoupling. Specifically, when the regulations get strengthening, foreign investors increase over-decoupling in the firms with high board centrality.

Third, our evidence suggests that changes of Chinese CSR regulation have an important impact on corporate decisions in CSR practices. We find that the relationship between board centrality and CSR decoupling endures significant changes because of the adoption of the 2015 new environment law. The findings add to the previous studies on Chinese CSR regulation policies (Zhang et al., 2017; Liu et al., 2020; Yu et al., 2021).

## Theoretical Background and Hypotheses

### Board Network and Corporate Practices

Existing literature based on network theory has shown that through direct and indirect connections within networks, network members can gain access to and share critical resources and information in time and enrich knowledge, which forms important social capital (Burt, 1987, 1992; Nahapiet and Ghoshal, 1998; Woolcock and Narayan, 2000). Because of the different positions of members in the networks, Adler and Kwon (2002) argue that the advantages conferred by one’s position within the networks can be converted to some advantages, and the degree of the position advantage is defined as network centrality.

Board of directors is an important part of the top management team; hence, its network plays a critical role in corporate practices. Firms with higher board network centrality have a higher ability to exchange and use information that allows them to make more effective decision-making than their peers. Existing literature indicates that firms with higher board centrality tend to have better access to finance (Larcker et al., 2013; Chuluun et al., 2014; Renneboog and Zhao, 2014; Rousseau and Stroup, 2015; Feng et al., 2019), greater performance in mergers and acquisitions (Renneboog and Zhao, 2014; Rousseau and Stroup, 2015), and better financial performance (Larcker et al., 2013).

Further studies argue that board networks have a significant impact on CSR. Due to the advantage of social capital accumulation, information access, and so on, Harjoto and Wang (2020) indicate that firms with higher board centrality have higher CSP. Similarly, Nandy et al. (2020) find that there is a positive relationship between director centrality^1^ and CSP by using listed firms from 17 countries, and this positive effect is more pronounced after the 2008 financial crisis. Lai et al. (2020) further complement the effect of corporate governance, institutional ownership, public awareness, and the high commitment of stakeholders on the relationship between board centrality and CSP. Moreover, in emerging market research, there is some indirect evidence supporting a positive relationship between board centrality and CSP. Li et al. (2019) find that the relationship between director network centrality and philanthropic donation is positive, and corporate donation is the most important part of discretionary components in CSR (Lin et al., 2015). Compared with CSP, the evidence for CSD is short, but some indirect evidence may support that a positive influence of board centrality on CSD. Muttakin et al. (2018) finds that board social capital is positively related to CSD, and high board centrality is positively related to social capital (Larcker et al., 2013). Evidence from Sun et al. (2020) find that board of interlocks, which is related to the concept of board centrality, has a positive influence on CSD in the Chinese market.

### Board Network and CSR Decoupling

The existing literature shows that CSR decoupling refers to the gap between internal and external actions in CSR practices (Tashman et al., 2019), specifically, CSP reflects corporate internal actions (Hinze and Sump, 2019) and CSD reflects corporate external actions (Dhaliwal et al., 2012). Therefore, CSR decoupling refers to the gap between CSP and CSD. CSR decoupling mainly includes two forms. First, firms decouple their commitment in the CSD from the actual CSP. Specifically, firm’s commitment to CSD does not match their CSP (Sauerwald and Su, 2019). Second, firms decouple their CSD level from the CSP level; that is, the CSD level provided in the annual report or CSR report is higher or lower than the level of actual CSP (Delmas and Burbano, 2011; García-Sánchez et al., 2020).

Existing literature explains the driving mechanism of CSR decoupling from different theoretical perspectives. Tashman et al. (2019) based on the neo-institutional theory, argue that institutional characteristics in different markets drive CSR decoupling of multinational enterprises. Based on the agency theory, Shahab et al. (2021) argue that more powerful CEOs are more short-sighted and have higher CSR decoupling in their firms; Parra-Domínguez et al. (2021) find that CSR decoupling is lower in family firms, because the family firms suffer lower agency cost. Based on the overconfidence theory, Sauerwald and Su (2019) find that managerial overconfidence increases CSR decoupling. Based on the information asymmetry theory, Zhang, (2021) finds that analyst coverage helps to alleviate the information asymmetry between stakeholders and firms, thereby reducing CSR decoupling. Similarly, Sánchez et al. (2021) find that assurance of CSR reports helps reduce information asymmetry, thus decreasing decoupling practices.

Regarding the influence of board network centrality on CSR decoupling, we build a theoretical framework mainly based on the information asymmetry theory (Myears and Majluf, 1984). Compared with actual CSP, when a firm has a higher CSD, there exists a positive gap in the firm. We define the positive gap as over-decoupling in the following. Specifically, firms implement symbolic management strategies in CSR practices (Walker and Wan, 2012; Tashman et al., 2019). Symbolic management refers to the fact that a firm’s actual practices do not conform to its espoused policies, resulting in misalignment between the two (Meyer and Rowan, 1977; Carpenter and Westphal, 2001; Fiss and Zajac, 2006).

We argue that firms with higher board network centrality widen the over-decoupling. First, boards with high centrality can expand the asymmetry information barrier between firms and their stakeholders. Specifically, boards with higher centrality have stronger power to influence public opinion, which means they can relieve the possible exposure risk of symbolic management. Especially in China, news media and other public opinion channels are subject to stronger restrictions (Wang et al., 2019). For example, Piotroski et al. (2014) indicates that Chinese politicians restrict and eliminate adverse news from firms with strong connections for their own interests. Therefore, in China, boards with higher centrality are more likely to use their network directly or indirectly to connect with political authority, intervening in news reports, social media and other public opinion systems, thereby weakening the exposure risks of symbolic management. Similar logic has also been found in other corporate practices; for example, firms with higher board centrality implement more inefficient mergers and acquisitions (Tao et al., 2019) and higher earnings management (Abdul Wahab et al., 2020), because they are more likely to circumvent the influence of public opinion supervision. Second, boards with higher centrality help to reduce information asymmetry among them and their connecting firm, making it easier for their firms to obtain and utilize information (Larcker et al., 2013), namely, allowing them to effectively observe and learn the successful experience of symbolic practices from other peers and then use them in their own firms (Nandy et al., 2020). Hence, we formulate hypothesis 1:

*Hypothesis 1*: Board network centrality increases CSR over-decoupling.

Another misalignment between CSD and CSP is the negative one. To be specifically, compared with the actual CSP, firms tend to have a lower CSD, which is defined as under-decoupling in this paper. The main reason for under-decoupling is that firms are worried about the incremental legitimacy pressure caused by a high level of CSD (Carlos and Lewis, 2018). When a firm discloses more information, it will attract more attention from stakeholders (Cormier and Magnan, 2014; Ji et al., 2015), which also provides evidence for external stakeholders (such as SRIs and green NGOs) in inquiries and lawsuits; thus, firms have to carefully decide the scope and accuracy of the CSD to prevent facing incremental pressure (Carlos and Lewis, 2018). Especially in China, firms’ motivations for CSR practices are more complicated, even some ones are dark (Qian and Chen, 2021), such as covering up firms’ political costs. Lin et al. (2015) and Jia and Zhang (2018) find that some Chinese firms engage in CSR practices in exchange for gaining more political connections. Therefore, these firms prefer to engage in CSR practices “silently” rather than attracting the attention of other stakeholders, which leads to a lower CSD (Marquis and Qian, 2014).

Based on the previous framework, we argue that board network centrality has no impact on under-decoupling. When a firm chooses a low CSD to circumvent stakeholders’ attention to its actual CSP, it has established an information asymmetry barrier for itself. In other words, without incremental disclosure, legitimacy threats related to CSD will disappear in the under-decoupling firms. In this case, the sensitivity of board centrality and decoupling should not exist. Hence, we formulate hypothesis 2:

*Hypothesis 2*: Board network centrality has no impact on CSR under-decoupling.

**Figure 1:**
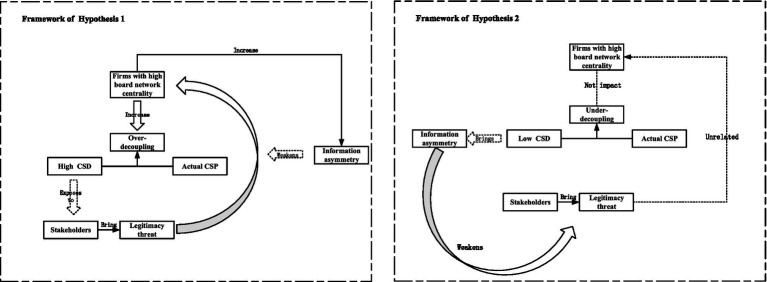
Theoretical framework.

Figure 1 reports the theoretical framework for our hypothesis development.

## Materials and Methods

### Data Sources

The original samples are Chinese A-share listed firms on the Shanghai and Shenzhen stock exchanges for the 2009–2018 period. We collect our data from multiple sources: (1) financial data of the capital market and firms are from CSMAR; (2) following Larcker et al. (2013), we calculate board centrality by Pajek, and the original data of corporate board is from CSMAR; (3) RKS rating comes from Rankings, and the available range is from 2009 to 2018. Therefore, our final sampling period is 2009 to 2018.

We perform the following preprocessing steps: (1) we exclude firms from the financial industry and firm-year observations with missing data; (2) to avoid the impact of extreme values, we winsorize all continuous variables at the 1 and 99% levels. After screening, our final sample consists of 5,729 firm-years.

### Measurements of Main Variables

#### Board Network Centrality

Referring to historical studies (El-Khatib et al., 2015; Larcker et al., 2013), this study creates a proxy of network centrality (Centrality) by using four commonly used measures; that is, degree centrality, closeness centrality, betweenness centrality, and eigenvector centrality. Specifically, we sort four centrality measures into 10 quantiles and then take the mean value of four processed variables as the proxy of board network centrality.

Degree centrality measures the number of direct connections between firms through sharing at least one board member. The more connections a firm has, the higher the centrality of the firm’s board network, and the stronger the firm’s ability to obtain information. Closeness centrality measures the firm’s closeness to other firms through the shortest connection and measures the firm’s efficiency in obtaining information from others through the board network. The closer the connections with other firms, the more information and resources will be transmitted through fewer firms, information and resources will be exchanged faster, more accurately and in more detail, and the quality of information will be higher. Betweenness centrality measures how often a firm sits at the shortest “bridge” position between the other two firms. If a firm sits on the shortest connection of multiple pairs of firms, then the firm plays a vital role in connecting firms and exchanging information and resources by promoting, obstructing or even changing the communication between other firms. Eigenvector centrality considers not only the number of directly connected firms but also the number of indirectly connected firms. In other words, if a firm’s directly connected firm has many connections, then the firm will also have higher connection accordingly, which means that it has more power to influence other firms in terms of information dissemination and exchange through these well-connected firms and enjoy more and more stable information flow and greater visibility.

#### CSR Decoupling

CSR decoupling refers to the misalignment between firms’ internal and external CSR actions (Tashman et al., 2019). Internal actions are firms’ real CSR practices, such as the inputs on donations and environmental protection and so on, and larger CSR inputs ultimately reflect higher CSP. External actions generally focus on communication and visible disclosure that firms adopt to create a good reputation in the views of the public, including the commitment and statement of CSR practices and so on, which reflects firm’s CSD level. There are three main types of definition for CSR decoupling: (1) the difference between a firm’s CSD level rated by third-party ratings and its actual CSR performance or inputs (García-Sánchez et al., 2020; Zhong et al., 2021); (2) the difference between internal (e.g., employee welfare expenditure) and external CSR actions (e.g., employee improvement commitment; Sánchez et al., 2021; Shahab et al., 2021); (3) the difference between level of optimistic tone from CSR reports and CSR performance (Sauerwald and Su, 2019; Zhang, 2021).

Considering the reality of the Chinese market, we refer to García-Sánchez et al. (2020), and define CSR decoupling as the difference between CSD and CSP. Following Liao et al. (2019), we use the technical dimension score (T-score) and content dimension score (C-score) provided by RKS ratings proxies for CSD and CSP.^2^ We normalized both CSD and CSP on the scale of [0, 1] to make these two variables comparable; after this, we obtain the decoupling variable as the difference between CSD and CSP. Then, according to the direction of CSR decoupling, the samples are divided into the following two groups. The positive difference indicates that a firm’s CSD level is higher than CSP level; that is, positive decoupling, defined as Gap_over. The economic meaning of Gap_over is that firms tend to use more CSD to improve their social reputation, instead of inputting more resources in actual practices to get better CSP. The negative difference indicates that a firm performs better than its disclosure, that is, negative decoupling, defined as Gap_under. The economic meaning of Gap_under is that firms input more resources in actual CSR practices, but lack of related CSD, because they are afraid they are exposing more to stakeholders (Kim and Lyon, 2015; Carlos and Lewis, 2018). To better understand, we refer to the calculation method of investment efficiency (Chen et al., 2011), and we take the absolute values of both CSR decoupling variables; the higher the value is, the larger the gap between CSD and CSP.

#### Control Variable

Following previous studies, e.g., Su (2019) and Wen and Song (2017), control variables are employed as follows: (1) resource abundance variables, resources controlled by firms and the ability to acquire resources are related to CSR engagement. CASH, equal to the logarithm of firm cash holdings. ROA, measured as the return-on-assets ratio. BTM, the book-to-market ratio. LEV, measured by the asset-liability ratio.

(2) Reputation variables, the visibility of firms in society is related to their CSR engagement. SIZE, equal to the logarithm of total assets, stands for the visibility of the firm and the political cost that the firm may face. AGE, measured as the natural logarithm of the firm listing period. (3) Corporate governance variables, firms with better governance have stronger motivations and mechanisms to engage in CSR. TOP1, defined by the percentage of stock held by a firm’s largest shareholder. MSH, measured as the percentage of stockholdings by top management team. IB, measured as the ratio of independent directors on the board. BSIZE, measured as the natural logarithm of the total number of directors. DUAL is a dummy variable; if one person is both CEO and chairman, the value is 1, otherwise 0. Finally, to control for variation across time and industry, we include year and industry dummies.

### Model Design

For Hypotheses 1 to 2, we design model (1):


(1) $$\begin{array}{l} \begin{array}{l} {\mathrm{CSP}}_{i,t}/{\mathrm{CSD}}_{i,t}/{\mathrm{Gap}}_{i,t}=\alpha_{0}+\beta_{1}{\mathrm{Centrality}}_{i,t-1}\\ \;+\sum \beta_{j}{\mathrm{Control variables}}_{i,t} \end{array}\\ \;\;\;\;\;+\sum \mathrm{Year}\&\mathrm{Indu effects}+\varepsilon_{i,t} \end{array}$$

where CSP and CSD represent CSP and disclosure, which equal to the scores of Content and Technicality dimension of RKS ratings; Gap represents CSR decoupling, specifically, we define three kinds of Gap, Gap_abs, which are equal to the absolute values of the positive gap and negative gap between CSD and CSP, Gap_over, which are equal to the absolute values of the positive gap, and Gap_under, which are equal to the absolute values of the negative gap; the larger the gap is, the higher the value. Centrality is our independent variable, which is equal to the mean value of four network centrality variables (degree, closeness, betweenness and eigenvector) after sorting them into 10 quantiles. Control variables refer to the set of control variables mentioned above. Finally, the year and industry effects are included in the regression. Following Petersen (2009), t-statistics are clustered at the firm and year level.

## Main Results

### Descriptive Statistics

Table 1 presents the descriptive statistics of our main variables used in the regression analysis of full samples of the full sample of 5,729 firm-year observations from 2009 to 2018. The mean value of the dependent variable Centrality is 6.13. The average scores for CSP and CSD, namely, C-score and T-score of RKS ratings,^3^ are 17.29 and 7.189, respectively. Furthermore, the mean value for key independent variables Gap_abs, Gap_over and Gap_under are .135, .117 and .144, respectively. For the control variables, our descriptive statistics are consistent with historical literatures. For examples, the mean values of ROA and LEV are .043 and .491 in our sample, which are consistent with Wen and Song (2017). The mean values of IB and BSIZE are .375 and 2.197, respectively, which are consistent with Su (2019). Further, the mean values of DUAL and AGE are consistent with Zhang (2021).

**Table 1:** Descriptive statistics.

Variable	Obs	Mean	Std. dev.	Min	P25	P50	P75	Max
Centrality	5,729	6.13	2.528	1	4.25	6.5	8.25	10
Corporate social performance (CSP)	5,729	17.29	5.871	6.15	13.18	16.35	20.39	35.51
Corporate social disclosure (CSD)	5,729	7.189	1.933	3.85	5.74	6.91	8.19	14.01
Gap_abs	5,729	.135	.095	0	.06	.12	.193	.567
Gap_over	2,045	.117	.089	0	.048	.099	.17	.567
Gap_under	3,684	.144	.096	0	.069	.131	.202	.553
CASH	5,729	21.01	1.466	15.1	19.99	20.92	21.91	26.49
ROA	5,729	.043	.061	−.854	.016	.037	.067	.482
BTM	5,729	.668	.253	.037	.474	.679	.87	1.43
LEV	5,729	.491	.201	.008	.343	.506	.643	1.513
SIZE	5,729	23.06	1.447	18.27	22.01	22.91	23.92	28.51
AGE	5,729	2.399	.643	0	2.079	2.565	2.89	3.367
TOP1	5,729	37.59	16.11	3	24.49	36.48	49.87	89.41
MSH	5,729	.029	.09	0	0	0	.003	.843
IB	5,729	.375	.059	.091	.333	.364	.4	.8
BSIZE	5,729	2.196	.21	1.386	2.079	2.197	2.303	2.89
DUAL	5,729	.176	.381	0	0	0	0	1

### Correlation Analysis

Table 2 presents the correlation coefficients for key variables in main analysis. The correlation between Centrality and CSP (CSD) is .181 (.216) at the 1% level. These results are consistent with the previous studies (e.g., Harjoto and Wang, 2020). Due to our design of decoupling variables, the samples between the variables Gap_over and Gap_under do not overlap with each other, accordingly, there is no correlation between them.

**Table 2:** Pearson’s correlation.

Variable	Centrality	CSP	CSD	Gap_abs	Gap_over	Gap_under	CASH	ROA	BTM	LEV	SIZE	AGE	TOP1	MSH	IB	BSIZE	DUAL
Centrality	1																
CSP	.181^***^	1															
CSD	.216^***^	.680^***^	1														
Gap_abs	−.030^**^	.163^***^	−.094^***^	1													
Gap_over	−.020	−.274^***^	.215^***^	1	1												
Gap_under	−.030^*^	.321^***^	−.194^***^	1	N/A	1											
CASH	.270^***^	.404^***^	.375^***^	.022^*^	−.057^**^	.055^***^	1										
ROA	−.018	.058^***^	−.003	.0170	−.062^***^	.051^***^	.064^***^	1									
BTM	.124^***^	.195^***^	.165^***^	.042^***^	.0200	.045^***^	.440^***^	−.277^***^	1								
LEV	.132^***^	.123^***^	.092^***^	.0170	−.052^**^	.050^***^	.350^***^	−.388^***^	.455^***^	1							
SIZE	.297^***^	.441^***^	.419^***^	.0170	−.058^***^	.048^***^	.869^***^	−.050^***^	.588^***^	.506^***^	1						
AGE	.187^***^	−.0002	.078^***^	−.0190	.071^***^	−.050^***^	.143^***^	−.152^***^	.125^***^	.241^***^	.230^***^	1					
TOP1	.066^***^	.165^***^	.072^***^	.0170	−.082^***^	.044^***^	.210^***^	.048^***^	.167^***^	.078^***^	.256^***^	−.072^***^	1				
MSH	−.139^***^	−.092^***^	−.052^***^	−.031^**^	−.001	−.038^**^	−.163^***^	.102^***^	−.148^***^	−.197^***^	−.231^***^	−.415^***^	−.099^***^	1			
IB	.0210	.028^**^	.050^***^	.001	.038^*^	−.0140	.122^***^	−.0140	.0190	.029^**^	.105^***^	−.027^**^	.076^***^	.049^***^	1		
BSIZE	.175^***^	.167^***^	.083^***^	.048^***^	−.054^**^	.088^***^	.166^***^	−.007	.151^***^	.113^***^	.223^***^	.075^***^	.023^*^	−.156^***^	−.398^***^	1	
DUAL	−.072^***^	−.065^***^	−.028^**^	−.00900	.0190	−.0150	−.069^***^	.061^***^	−.119^***^	−.105^***^	−.110^***^	−.173^***^	−.100^***^	.390^***^	.092^***^	−.159^***^	1

* *Indicates significance at the levels of 10%*;

** *Indicates significance at the levels of 5%*;

*** *Indicates significance at the levels of 1%*.

### Regression Results

The Chinese legislature carried out a major amendment to the Environmental Protection Law of China (referred to as “the new environmental law”) in April 2014, which significantly enhanced the law enforcement authority of environmental protection departments, expanded and strengthened the scope and quality of mandatory information disclosure (Zhang et al., 2017), it has become an important signal of the improvement of the Chinese CSR regulatory system (Yu et al., 2021). For example, since 2015, the number of Chinese institutional investors, NGOs and related practices based on CSR (ESG) themes has increased dramatically. According to Syntao. (2021), the number of public funds for ESG theme increases rapidly. Changes in the regulatory system cause fundamental changes that may affect stakeholder pressure for symbolic practices (Marquis et al., 2016). These changes remind us that it is better to conduct an analysis of different periods. Thus, in the regression of model (1), except for using the whole sample, we also run regressions with two separating subgroups: the pre-adoption group and post-adoption group of the new environmental law. We define a dummy variable Post, which equals 0 if the firm-years belong to 2009 to 2014, or 1 during 2015 to 2018.^4^

Table 3 reports the regression results of model (1). Columns (1) to (3) show the coefficients between Centrality and performance variable CSP are all positive, specifically, .0325 (*p* < .05) in the whole period, .0307 (not significant) and .0296 (*p* < .05) in the pre-adoption and post-adoption periods, respectively. The coefficients indicate that board network centrality is positively related to CSP, and this positive relationship is more pronounced in the post-adoption period of the new environmental law. Columns (4) to (6) show the regression results for the disclosure variable CSD. Similar to the results of CSP, the coefficients of Centrality are all positive, but more pronounced in the post-adoption period. The above evidence is consistent with previous research, e.g., Harjoto and Wang (2020), which finds that board centrality increases CSR. The more pronounced coefficients of Centrality in the post-adoption period suggest that it’s necessary to take into consideration of the influence of the new environmental law.

**Table 3:** Regression results of board network centrality and CSP/CSD/Gap.

Variable	CSP	CSP	CSP	CSD	CSD	CSD	Gap_over	Gap_over	Gap_over	Gap_under	Gap_under	Gap_under
	All	Post = 0	Post = 1	All	Post = 0	Post = 1	All	Post = 0	Post = 1	All	Post = 0	Post = 1
	(2009–2018)	(2009–2014)	(2015–2018)	(2009–2018)	(2009–2014)	(2015–2018)	(2009–2018)	(2009–2014)	(2015–2018)	(2009–2018)	(2009–2014)	(2015–2018)
	(1)	(2)	(3)	(4)	(5)	(6)	(7)	(8)	(9)	(10)	(11)	(12)
Centrality	.0325^**^	.0307	.0296^**^	.1389^**^	.1127	.1854^***^	−.0014	.0023^**^	−.0026^**^	−.0002	−.0007	.0020^*^
	(2.0707)	(1.3087)	(2.2038)	(2.5653)	(1.5747)	(3.6060)	(−1.3923)	(2.1132)	(−2.4718)	(−.1793)	(−.6720)	(1.8577)
CASH	.0786	.1006	.0289	.2811	.2172	.2438	.0018	.0027	.0021	.0026	.0017	.0062^**^
	(1.4183)	(1.3535)	(.5948)	(1.5184)	(.9801)	(1.2389)	(.7387)	(.3775)	(.7158)	(.8251)	(.4309)	(2.1120)
ROA	−1.6256^*^	−3.1231^***^	−.5692	−.7074	−3.0923	.1460	−.0844^*^	−.1678^***^	−.0414	.0967^**^	.1490^***^	.0120
	(−1.9465)	(−3.7324)	(−.8249)	(−.2839)	(−1.0783)	(.0566)	(−1.7729)	(−2.7163)	(−.7735)	(2.1558)	(3.0915)	(.3293)
BTM	−.9321^***^	−.9322^***^	−1.0363^***^	−2.3657^***^	−2.6502^***^	−2.6169^***^	−.0069	−.0107	.0032	.0031	.0038	.0111
	(−4.0178)	(−3.0648)	(−3.9455)	(−3.0211)	(−2.6552)	(−2.9372)	(−.6555)	(−.7212)	(.2375)	(.3987)	(.2992)	(.6912)
LEV	−.7394^**^	−1.0488^***^	−.4459^*^	−2.0850^**^	−2.3149^**^	−1.7441^*^	−.0065	−.0183	.0035	.0228	.0374^**^	−.0033
	(−2.4698)	(−2.9267)	(−1.7813)	(−2.2736)	(−2.0905)	(−1.7998)	(−.4223)	(−.6682)	(.1921)	(1.5502)	(2.2407)	(−.2697)
SIZE	.5592^***^	.5919^***^	.5878^***^	1.9497^***^	1.9197^***^	2.1747^***^	−.0067	.0002	−.0120^***^	.0021	.0024	−.0024
	(6.9301)	(5.7687)	(8.4169)	(7.7247)	(6.4835)	(8.5415)	(−1.4266)	(.0406)	(−2.5943)	(.5839)	(.4942)	(−.6729)
AGE	−.2840^***^	−.2945^***^	−.2667^***^	−.9411^***^	−1.0584^***^	−.9276^***^	−.0001	.0009	.0023	−.0059	−.0074	−.0028^***^
	(−3.9662)	(−3.0448)	(−4.2588)	(−3.8022)	(−3.6343)	(−3.4387)	(−.0328)	(.1103)	(.5505)	(−1.5799)	(−1.6101)	(−3.9459)
TOP1	.0018	.0017	.0016	.0185^*^	.0141	.0232^**^	−.0003^*^	−.0001	−.0003	.0001	.0001	.0001
	(.6158)	(.4560)	(.5791)	(1.7803)	(1.2649)	(2.1008)	(−1.8667)	(−.3941)	(−1.5915)	(.6621)	(.9277)	(.4107)
MSH	.5605	.6771	.4685	.0613	.1142	.0472	−.0246	−.0051	−.0323	−.0570^**^	−.0604^***^	−.0346
	(1.4724)	(1.2804)	(1.1261)	(.0484)	(.0708)	(.0303)	(−1.3245)	(−.0947)	(−1.5063)	(−2.4249)	(−2.5910)	(−.6153)
IB	.6465	−.0903	1.0953	.7474	.3735	1.1776	.0876^*^	.0699	.0677	.0056	.0250	−.0170
	(.8608)	(−.1067)	(1.5108)	(.3093)	(.1491)	(.3526)	(1.9284)	(1.3908)	(1.1298)	(.1355)	(.6194)	(−.2108)
BSIZE	.4477^**^	.4573	.3795^*^	2.0570^***^	2.2619^***^	1.7481^*^	.0116	.0257	.0100	.0322^***^	.0388^***^	.0159
	(2.0214)	(1.4536)	(1.9351)	(2.6623)	(2.5776)	(1.9348)	(.8553)	(1.2741)	(.5367)	(3.1221)	(3.0136)	(1.2512)
DUAL	−.1682^**^	−.1445	−.1655^*^	−.4316	−.1147	−.7484^**^	−.0025	.0042	−.0039	.0010	.0021	−.0024
	(−2.1223)	(−1.3356)	(−1.7789)	(−1.3001)	(−.2629)	(−2.0043)	(−.5144)	(.2937)	(−.8124)	(.1428)	(.2421)	(−.2767)
Constant	−8.2256^***^	−8.5891^***^	−6.8855^***^	−36.3719^***^	−36.2787^***^	−41.8466^***^	.1455	−.0811	.3323^***^	−.0892	−.1043	−.0270
	(−6.3981	(−5.4065)	(−5.4962)	(−8.5858)	(−7.6539)	(−10.0731)	(1.5762)	(−.8968)	(3.9220)	(−1.5054)	(−1.5979)	(−.3755)
Year	Yes	Yes	Yes	Yes	Yes	Yes	Yes	Yes	Yes	Yes	Yes	Yes
Industry	Yes	Yes	Yes	Yes	Yes	Yes	Yes	Yes	Yes	Yes	Yes	Yes
*N*	5,729	3,026	2,703	5,729	3,026	2,703	2045	455	1,590	3,684	2,571	1,113
*R* ^2^	.4075	.2645	.3333	.2934	.2954	.3163	.1794	.1961	.1656	.1640	.1480	.1842

* *Indicates significance at the levels of 10%*;

** *Indicates significance at the levels of 5%*;

*** *Indicates significance at the levels of 1%*.

For CSR decoupling, columns (7) to (9) report the regression results for the over-decoupling variable Gap_over. The coefficients on Centrality are complex. The coefficient is not significant in the whole period. However, the coefficients are .0023 (*p* < .05) in the pre-adoption period but −.0026 (*p* < .05) in the post-adoption period. The coefficient in column (8) partly supports Hypothesis 1, indicating that board centrality is positively related to over-decoupling, but only when CSR institutional regulation is weak. However, when institutional regulation strengthened after 2015, the relationship became negative. Columns (10) to (12) show the regression results for under-decoupling variable Gap_under. The results are as complex as Gap_over. We find that the coefficients on Centrality in the whole and post-adoption periods are not significant, supporting Hypothesis 2. However, in the post-adoption period, the coefficient is .0020 (*p* < .10), which supports our corner about the regulation change, namely, due to the enhanced supervision by the new environmental law, firms with high board centrality furtherly decrease their CSD, resulting in larger under-decoupling.

During the post-adoption period, centrality is negatively (positively) related to over-decoupling (under-decoupling). Our explanation is that boards with high centrality have higher information acquisition and utilization efficiency (Harjoto and Wang, 2020; D. Larcker et al., 2013), and they can understand institutional policy changes and related impacts more easily and deeply, then adjust corresponding strategies. Specifically, the new environmental law provides a more favorable foundation for stakeholders’ rights protection and supervision from CSR information (Zhang et al., 2017). As a reaction to the law, over-decoupling firms with high board centrality are more easily to notice the increasing cost of symbolic management, thereby reducing symbolic management. Meanwhile, under-decoupling firms with high board centrality can further strengthen the original information strategy, revealing less information and widening information asymmetry.

### Robustness Checks

#### Test Based on Original Centrality

In the main analysis, we use the mean value of four board network centrality variables (degree, closeness, betweenness and eigenvector) after sorting them into 10 quantiles as a proxy of board network centrality. In this analysis, we directly use four original network centrality variables as an alternative measurement for board centrality for robustness checks to ensure the robustness of the results. For brevity, we only report the results of the decoupling variable Gap_over and Gap_under, and the coefficients on control variables are omitted.

Table 4 reports the regression results based on four network centrality variables. The dependent variables in columns (1) to (8) are the over-decoupling variable Gap_over. The coefficients on centrality variables degree, closeness, betweenness and eigenvector are mostly positive (except for eigenvector) but not significant in the pre-adoption period, meanwhile, three of four coefficients are significantly negative in the post-adoption period (except for betweenness). Columns (9) to (16) show the results for Gap_under. The coefficients on degree, closeness, betweeness and eigenvector are all negative in the pre-adoption period and positive in the post-adoption period. The regression results above are similar to our main analysis results.

**Table 4:** Alternative measurements of board network centrality.

Variable	Gap_over	Gap_over	Gap_over	Gap_over	Gap_over	Gap_over	Gap_over	Gap_over	Gap_under	Gap_under	Gap_under	Gap_under	Gap_under	Gap_under	Gap_under	Gap_under
	Post = 0	Post = 0	Post = 0	Post = 0	Post = 1	Post = 1	Post = 1	Post = 1	Post = 0	Post = 0	Post = 0	Post = 0	Post = 1	Post = 1	Post = 1	Post = 1
	(2009–2014)	(2009–2014)	(2009–2014)	(2009–2014)	(2015–2018)	(2015–2018)	(2015–2018)	(2015–2018)	(2009–2014)	(2009–2014)	(2009–2014)	(2009–2014)	(2015–2018)	(2015–2018)	(2015–2018)	(2015–2018)
	(1)	(2)	(3)	(4)	(5)	(6)	(7)	(8)	(9)	(10)	(11)	(12)	(13)	(14)	(15)	(16)
Degree	.0023				−.0012^**^				−.0004				.0008			
	(1.5497)				(−2.5744)				(−.7464)				(1.3153)			
Closeness		.0557				−.1647^***^				−.0116				.0701		
		(1.4171)				(−4.8710)				(−.2141)				(1.4805)		
Betweenness			2.4563				−.5721				−1.1878^**^				1.6874	
			(1.4812)				(−.7008)				(−2.0294)				(.8730)	
Eigen vector				−3.3313				−7.7697^***^				−.6048				3.6448
				(−.7523)				(−4.3980)				(−.2854)				(.8003)
Constant	−.0691	−.0915	−.0663	−.0944	.3324^***^	.3493^***^	.3485^***^	.3485^***^	−.1014	−.0953	−.1120^*^	−.0950	−.0064	−.0194	−.0023	−.0155
	(−.9544)	(−1.1838)	(−.9009)	(−1.2118)	(4.2523)	(4.3461)	(4.5838)	(4.4711)	(−1.5289)	(−1.4795)	(−1.6969)	(−1.4467)	(−.0668)	(−.1893)	(−.0250)	(−.1539)
Control variables	Yes	Yes	Yes	Yes	Yes	Yes	Yes	Yes	Yes	Yes	Yes	Yes	Yes	Yes	Yes	Yes
Year	Yes	Yes	Yes	Yes	Yes	Yes	Yes	Yes	Yes	Yes	Yes	Yes	Yes	Yes	Yes	Yes
Industry	Yes	Yes	Yes	Yes	Yes	Yes	Yes	Yes	Yes	Yes	Yes	Yes	Yes	Yes	Yes	Yes
*N*	455	455	455	455	1,590	1,590	1,590	1,590	2,571	2,571	2,571	2,571	1,113	1,113	1,113	1,113
R^2^	.1996	.1913	.1978	.1909	.1647	.1665	.1625	.1647	.1479	.1477	.1486	.1477	.1832	.1827	.1830	.1827

* *Indicates significance at the levels of 10%*;

** *Indicates significance at the levels of 5%*;

*** *Indicates significance at the levels of 1%*.

#### Heckman Approach

Among the 24,000 more original observations in the research period from 2009 to 2018, only approximately 24% of the observations with CSD are included in our main analysis, which may cause sample selection bias. For this problem, we use the Heckman method (Heckman, 1979). In the first step, we design the probit model as follows:


(2) $$\begin{array}{l} \mathrm{probit}\left(\mathrm{CSD}\_{\mathrm{dummy}}_{i,t}\right)=\alpha_{0}+\sum \beta_{j}\mathrm{CSD}\;{\mathrm{Determinates}}_{i,t}\\ \;+\sum \mathrm{Year}\&\mathrm{Indu effects}+\varepsilon_{i,t} \end{array}$$

where CSD_dummy is a dummy variable. If the firm discloses CSR information, the value is 1; otherwise, it is 0. Following Li et al. (2013), the influential variables of CSD are as follows: (1) ROA, the return on firm equity; (2) SOE, is a dummy variable, if firm is state-owned, the value is 1, otherwise 0; (3) SIZE, is measured as the natural logarithm of total assets; (4) AGE, is measured as the natural logarithm of firm listing period; (5) LEV, is measured as asset-to-liability ratio; (6) TOP1, is measured as the percentage of stockholdings by the largest shareholder; (7) Herfindahl 5, is measured as the degree of ownership dispersion, calculated as Herfindahl–Hirschman Index of stockholdings by top five shareholders; (8) MSH, is measured as the percentage of stockholdings by top management team; (9) Year and Industry effects. Column (1) of Table 5 reports the result from estimating model (2).

**Table 5:** Results of Heckman’s approach.

Variable	Probit regression	Heckman two-stage estimation: second stage							
	CSD_dummy	CSP	CSP	CSD	CSD	Gap_over	Gap_over	Gap_under	Gap_under
	Original samples	Post = 0	Post = 1	Post = 0	Post = 1	Post = 0	Post = 1	Post = 0	Post = 1
	(2009–2018)	(2009–2014)	(2015–2018)	(2009–2014)	(2015–2018)	(2009–2014)	(2015–2018)	(2009–2014)	(2015–2018)
	(1)	(2)	(3)	(4)	(5)	(6)	(7)	(8)	(9)
Centrality		.1239^*^	.1841^***^	.0386	.0291^**^	.0024^**^	−.0026^**^	−.0008	.0020
		(1.6951)	(3.6719)	(1.6421)	(2.2096)	(2.4094)	(−2.3441)	(−.7809)	(1.5307)
ROA	1.3655^***^	1.3836	3.5636	−.2893	1.0833	−.0911	−.0471	.1095^**^	.0366
	(6.7186)	(.4578)	(1.4141)	(−.3312)	(1.4102)	(−1.3417)	(−.8528)	(2.0163)	(.5868)
SOE	.2599^***^								
	(10.2821)								
SIZE	.5827^***^	3.3142^***^	3.2436^***^	1.4273^***^	1.0832^***^	.0314^*^	−.0152	−.0067	.0070
	(51.0688)	(4.8488)	(5.0575)	(6.2442)	(5.4154)	(1.8528)	(−.9471)	(−.7267)	(.5935)
AGE	.2585^***^	−.2320	−.3476	.2097	.0306	.0181	.0012	−.0135^*^	.0019
	(15.7031)	(−.5248)	(−.8058)	(1.5464)	(.2642)	(1.3092)	(.1442)	(−1.9497)	(.2247)
LEV	−.7854^***^	−4.0045^***^	−2.9890^**^	−2.0485^***^	−1.0289^***^	−.0619	.0074	.0480^**^	−.0134
	(−11.7523)	(−3.2138)	(−2.2851)	(−4.8080)	(−3.2725)	(−1.4815)	(.2532)	(2.4920)	(−.5552)
TOP1	−.0033	.0167	.0274^**^	.0028	.0032	−.0001	−.0003^*^	.0001	.0001
	(−1.3303)	(1.4894)	(2.4687)	(.7507)	(1.0271)	(−.3502)	(−1.6476)	(.9099)	(.3982)
Herfindahl_5	.3142								
	(.9883)								
MSH	−.2266^**^	−2.0322	−1.1292	−.5986	−.0656	−.0378	−.0286	−.0429^**^	−.0507
	(−2.2565)	(−1.2531)	(−.6923)	(−1.1595)	(−.1303)	(−.5916)	(−1.1255)	(−2.1332)	(−1.2815)
CASH		.1983	.2191	.0918	.0266	.0002	.0023	.0015	.0064
		(.9626)	(1.1700)	(1.3045)	(.5304)	(.0302)	(.7445)	(.3634)	(1.4798)
BTM		−2.3543^**^	−2.2693^**^	−.7458^**^	−.8997^***^	−.0017	.0029	.0001	.0101
		(−2.4511)	(−2.5658)	(−2.5049)	(−3.6013)	(−.1176)	(.2249)	(.0084)	(.5678)
IB		.6117	1.6767	−.0428	1.2212	.0787	.0657	.0270	−.0191
		(.2375)	(.4908)	(−.0491)	(1.6077)	(1.6451)	(1.0900)	(.6832)	(−.3012)
BSIZE		2.5491^***^	1.9460^**^	.6269^**^	.4708^**^	.0303	.0095	.0366^***^	.0164
		(2.9248)	(2.1235)	(2.0316)	(2.2514)	(1.5297)	(.5313)	(2.7406)	(1.0867)
DUAL		−.1496	−.8089^**^	−.1597	−.1908^**^	.0043	−.0036	.0021	−.0022
		(−.3348)	(−2.1764)	(−1.4547)	(−2.1394)	(.3166)	(−.7335)	(.2483)	(−.2536)
Lambda		3.9883^**^	2.9016^*^	2.4108^***^	1.4003^***^	.0771^*^	−.0071	−.0284	.0260
		(2.2448)	(1.9502)	(4.5965)	(2.9445)	(1.9414)	(−.1948)	(−1.3773)	(.8430)
Constant	−13.8257^***^	−70.8220^***^	−70.2524^***^	−31.1217^***^	−20.2923^***^	−.8546^**^	.4091	.1478	−.2771
	(−53.3399)	(−3.9268)	(−4.4697)	(−5.5885)	(−3.9402)	(−2.2338)	(1.0494)	(.6601)	(−.9183)
Year	Yes	Yes	Yes	Yes	Yes	Yes	Yes	Yes	Yes
Industry	Yes	Yes	Yes	Yes	Yes	Yes	Yes	Yes	Yes
*N*	24,608	3,026	2,703	3,026	2,703	455	1,590	2,571	1,113
*R* ^2^		.2911	.3080	.2686	.3262	.2064	.1656	.1489	.1851
Pseudo *R*^2^	.2447								

* *Indicates significance at the levels of 10%*;

** *Indicates significance at the levels of 5%*;

*** *Indicates significance at the levels of 1%*.

Second, we regress based on model (1); meanwhile, the control variables also include the inverse Mills ratio calculated in the first step. Columns (2) to (9) of Table 5 show the results of model (1). The dependent variables in columns (2) to (3), columns (4) to (5), columns (6) to (7) and columns (8) to (9) are CSP, CSD Gap_over and Gap_under, respectively. For brevity, we only report the coefficients on Centrality, respectively, in the pre-adoption and post-adoption period. The results are similar to our main analysis results.

#### Endogeneity Test

The relationship between board network centrality and CSR practices investigated in this paper may be affected by other unobservable factors, which may lead to endogeneity problems. Therefore, the two-stage least squares (2SLS) approach is adopted to solve the problem of endogeneity. In the 2SLS estimations, the instrumental variable Centrality_IV is used, which defined as the centrality level in year t + 1.^5^ For brevity, we do not report the results in the whole periods, and the coefficients on control variables are omitted.

Table 6 shows the results of instrumental regression. Columns (1) to (4), columns (5) to (8), columns (9) to (12) and columns (13) to (16) report the results of CSP, CSD, Gap_over and Gap_under, respectively. The coefficients of CSP, CSD, Gap_over and Gap_under in the 2SLS approach are basically consistent with the main analysis.

**Table 6:** Results of 2SLS approach.

Variable	Centrality	CSP	Centrality	CSP	Centrality	CSD	Centrality	CSD	Centrality	Gap_over	Centrality	Gap_over	Centrality	Gap_under	Centrality	Gap_under
	Post = 0	Post = 0	Post = 1	Post = 1	Post = 0	Post = 0	Post = 1	Post = 1	Post = 0	Post = 0	Post = 1	Post = 1	Post = 0	Post = 0	Post = 1	Post = 1
	(2009–2014)	(2009–2014)	(2015–2018)	(2015–2018)	(2009–2014)	(2009–2014)	(2015–2018)	(2015–2018)	(2009–2014)	(2009–2014)	(2015–2018)	(2015–2018)	(2009–2014)	(2009–2014)	(2015–2018)	(2015–2018)
	First	Second	First	Second	First	Second	First	Second	First	Second	First	Second	First	Second	First	Second
	(1)	(2)	(3)	(4)	(5)	(6)	(7)	(8)	(9)	(10)	(11)	(12)	(13)	(14)	(15)	(16)
Centrality _IV	.595^***^		.495^***^		.595^***^		.495^***^		.638^***^		.493^***^		.584^***^		.497^***^	
	(39.09)		(18.91)		(39.09)		(18.91)		(16.84)		(13.36)		(34.97)		(13.15)	
Centrality		.258^***^		.256^*^		.057^***^		.035		.002		−.004		.002		.008^***^
		(4.02)		(1.84)		(2.70)		(1.00)		(1.01)		(−1.42)		(1.49)		(2.63)
Constant	−4.359^***^	−28.034^***^	−4.945^***^	−31.512^***^	−4.359^***^	−7.372^***^	−4.945^***^	−6.801^***^	−1.960	−.040	−4.187^*^	.102	−4.282^***^	−.085^**^	−5.555^***^	−.038
	(−5.50)	(−13.67)	(−3.29)	(−7.73)	(−5.50)	(−1.99)	(−3.29)	(−6.67)	(−.99)	(−.54)	(−1.93)	(1.14)	(−4.86)	(−2.04)	(−2.60)	(−.43)
Control variables	Yes	Yes	Yes	Yes	Yes	Yes	Yes	Yes	Yes	Yes	Yes	Yes	Yes	Yes	Yes	Yes
Year	Yes	Yes	Yes	Yes	Yes	Yes	Yes	Yes	Yes	Yes	Yes	Yes	Yes	Yes	Yes	Yes
Industry	Yes	Yes	Yes	Yes	Yes	Yes	Yes	Yes	Yes	Yes	Yes	Yes	Yes	Yes	Yes	Yes
*N*	3,023	3,023	1,267	1,267	3,023	3,023	1,267	1,267	453	453	659	659	2,570	2,570	608	608
*R* ^2^	.442	.244	.323	.216	.442	.217	.323	.263	.498	.050	.334	.047	.430	.092	.319	.010

* *Indicates significance at the levels of 10%*;

** *Indicates significance at the levels of 5%*;

*** *Indicates significance at the levels of 1%*.

#### Alternative Explanation of Political Connection

Existing research argues that CSR practices are significantly affected by political connections in the Chinese market (Li et al., 2015; Lin et al., 2015; Wang et al., 2020). Therefore, for the results in the main analysis, another alternative explanation is that the relationship between board centrality and CSR practices may be caused by political connections. To testing of this argument, we design the following exclusion tests:

First, we add the control variable politically connections (PC) into regression model (1), which equals 1 if a firm’s CEO or chairman who is a former government official (served in government agencies at or above the county level, the municipal people’s congress, or the army), and 0 otherwise. If we add the control variable of PC into the regression and the coefficients on Centrality lost significance, these should support the political explanation. Second, we design an alternative sample excluding political-related firms and only use non-political-related firms for regression. If we find that the coefficients on Centrality lost significance in the test with the alternative sample, the political explanation should hold. For brevity, we only report the results in post-adoption period.

The regression results are shown in Table 7. The results of adding the PC variable are reported in columns (1) to (4), and results of the alternative sample design are reported in column (5) to (8), the coefficients on Centrality are basically consistent with the main analysis. The alternative explanation of political connection does not hold.

**Table 7:** Alternative explanation of political connections.

Variable	CSP	CSD	Gap_over	Gap_under	CSP	CSD	Gap_over	Gap_under
	Post = 1	Post = 1	Post = 1	Post = 1	Post = 1 &PC = 0	Post = 1 &PC = 0	Post = 1 &PC = 0	Post = 1 &PC = 0
	(2015–2018)	(2015–2018)	(2015–2018)	(2015–2018)	(2015–2018)	(2015–2018)	(2015–2018)	(2015–2018)
	(1)	(2)	(3)	(4)	(5)	(6)	(7)	(8)
Centrality	.1853^***^	.0293^**^	−.0026^**^	.0020^*^	.2481^***^	.0322^*^	−.0022	.0040^**^
	(3.6172)	(2.1840)	(−2.4152)	(1.8485)	(4.0316)	(1.8684)	(−1.5696)	(2.4329)
PC	−.0208	−.0711	−.0099^**^	−.0032				
	(−.0686)	(−.7922)	(−2.0494)	(−.5563)				
CASH	.2433	.0271	.0020	.0061^**^	.3165	.0662	.0024	.0022
	(1.2427)	(.5672)	(.6892)	(2.1453)	(1.1570)	(.9777)	(.4167)	(.2251)
ROA	.1536	−.5430	−.0367	.0119	4.3343	.0355	−.0803	−.0051
	(.0592)	(−.8025)	(−.6901)	(.3173)	(1.2617)	(.0531)	(−1.2576)	(−.0961)
BTM	−2.6216^***^	−1.0523^***^	.0010	.0102	−2.0148^*^	−.7500^***^	−.0011	−.0222
	(−2.9680)	(−3.9835)	(.0755)	(.6453)	(−1.7667)	(−3.3446)	(−.0636)	(−.8396)
LEV	−1.7455^*^	−.4507^*^	.0036	−.0039	−1.2507	−.4082	.0060	.0034
	(−1.8038)	(−1.8096)	(.2048)	(−.3290)	(−.9840)	(−1.3602)	(.2673)	(.2410)
SIZE	2.1763^***^	.5933^***^	−.0116^**^	−.0019	1.8985^***^	.5251^***^	−.0098^**^	.0034
	(8.6106)	(8.5014)	(−2.4865)	(−.6098)	(5.3382)	(5.4497)	(−2.3340)	(.3137)
AGE	−.9279^***^	−.2676^***^	.0024	−.0030^***^	−.6254^*^	−.2157^**^	−.0022	−.0062
	(−3.4320)	(−4.2854)	(.5997)	(−4.4153)	(−1.6970)	(−2.5102)	(−.3742)	(−1.3339)
TOP1	.0232^**^	.0015	−.0003	.0001	.0351^**^	−.0001	−.0008^***^	.0002
	(2.0922)	(.5309)	(−1.6453)	(.3264)	(2.1232)	(−.0376)	(−2.8476)	(.8836)
MSH	.0559	.4982	−.0274	−.0330	.6007	.4113	−.0891^*^	−.0671
	(.0355)	(1.2128)	(−1.3638)	(−.5834)	(.2986)	(.8245)	(−1.7910)	(−.8912)
IB	1.1858	1.1234	.0713	−.0156	.9493	.9438	.1356	.0832
	(.3541)	(1.5447)	(1.1836)	(−.1922)	(.2163)	(.9310)	(1.5465)	(.8955)
BSIZE	1.7502^*^	.3867^**^	.0109	.0164	1.8646^*^	.4752^*^	.0105	.0201
	(1.9321)	(1.9706)	(.5805)	(1.3408)	(1.9239)	(1.8299)	(.7351)	(1.3305)
DUAL	−.7484^**^	−.1653^*^	−.0036	−.0025	−1.0671^**^	−.1320	.0142^*^	.0061
	(−2.0042)	(−1.7802)	(−.7807)	(−.2850)	(−2.2496)	(−1.2741)	(1.7958)	(.5155)
Constant	−41.8661^***^	−6.9523^***^	.3272^***^	−.0330	−39.7202^***^	−6.0534^***^	.2853^***^	−.1306
	(−10.0107)	(−5.4801)	(3.7680)	(−.4649)	(−7.9281)	(−4.4579)	(4.7605)	(−1.4525)
Year	Yes	Yes	Yes	Yes	Yes	Yes	Yes	Yes
Industry	Yes	Yes	Yes	Yes	Yes	Yes	Yes	Yes
*N*	2,703	2,703	1,590	1,113	1,581	1,581	952	629
*R* ^2^	.3163	.3336	.1682	.1844	.3219	.3432	.2036	.2084

* *Indicates significance at the levels of 10%*;

** *Indicates significance at the levels of 5%*;

*** *Indicates significance at the levels of 1%*.

## Additional Test

### Heterogeneity Test of Regional Environment Regulation

In the hypotheses development, we argue that one of the paths for the influence of board network centrality on CSR decoupling is through circumventing the public opinion supervision. Existing literature suggests that there is a strong relationship between public opinion supervision and regional environment regulation (Ruiqian and Ramakrishnan, 2018; Sun et al., 2019). Firms face stronger supervision in regions with higher degree of environmental regulation, resulting in lower over-decoupling and higher under-decoupling level. If higher board centrality can circumvent the influence of public opinion supervision, then the negative (positive) relationship between regulation and over-decoupling (under-decoupling) should be mitigated.

Referring to Xie et al. (2017) and Ruiqian and Ramakrishnan (2018), we use the number of environmental administrative penalty cases as the proxy of the level of regional environment regulation. We define variable Regulation, measured as the logarithm of the number of province environmental administrative penalty cases.^6^ We design an interaction model as follows:


(3) $$\begin{array}{l} \begin{array}{l} \mathrm{Gap}\_{\mathrm{over}}_{i,t}/\mathrm{Gap}\_{\mathrm{under}}_{i,t}=\alpha_{0}+\beta_{1}{\mathrm{Centrality}}_{i,t-1}\\ \;\;\times {\mathrm{Regulation}}_{i,t-1} \end{array}\\ \begin{array}{l} \;\;+\beta_{2}{\mathrm{Centrallity}}_{i,t-1}\\ \;\;+\beta_{3}{\mathrm{Regulation}}_{i,t-1}\; \end{array}\\ \begin{array}{l} \;\;+\sum {\mathrm{Control variables}}_{i,t}\\ \;\;+\sum \mathrm{Year}\&\mathrm{Indu effects}+\varepsilon_{i,t} \end{array} \end{array}$$

where the dependent variables Gap_over/Gap_under represent over-decoupling and under-decoupling, respectively. Regulation is a proxy for the level of regional environment regulation. Centrality×Regulation is the interaction term between the regulation variable and the centrality variable, which is our main interest. Control variables, year and industry effects are consistent with those of model (1).

Table 8 shows the regression results. For brevity, the coefficients on control variables are omitted. First, we use variable Regulation as the dependent variable. To be specific, columns (1), (3), and (5) report the regression results for over-decoupling variable Gap_over, and column (7), (9), and (11) shows the results for the under-decoupling variable Gap_under based on different periods. As shown in column (3) and (9), we find that the variable Regulation is significantly and negatively related (positively correlated) to Gap_over (Gap_under) in the pre-adoption period, but this relationship disappear in the post-adoption period. Before the implementation of the new environmental law, firms in regions with stronger environmental regulations face more stringent CSR supervision, resulting in a decrease of over-decoupling and an increase of under-decoupling. However, after the implementation of the new environmental law, all firms face stronger supervision, and the sensitivity of CSR to environmental regulations disappears. Moreover, we use the interaction effect model (3) for regression, and the regression results for variables Gap_over and Gap_under based on different periods are reported in columns (2), (4), (6) and columns (8), (10), (12), respectively. Specifically, the coefficients between Centrality×Regulation and Gap_over are positive but not significant. Then, the coefficients between Centrality×Regulation and Gap_under are negative, and are both significant at the 1% level. The evidence above suggests that board network centrality helps firms circumvent the influence of public opinion supervision, which is consistent with our theoretical expectations.

**Table 8:** Heterogeneity test of environmental regulation.

Variable	Gap_over	Gap_over	Gap_over	Gap_over	Gap_over	Gap_over	Gap_under	Gap_under	Gap_under	Gap_under	Gap_under	Gap_under
	All	All	Post = 0	Post = 0	Post = 1	Post = 1	All	All	Post = 0	Post = 0	Post = 1	Post = 1
	(2009–2018)	(2009–2018)	(2009–2014)	(2009–2014)	(2015–2018)	(2015–2018)	(2009–2018)	(2009–2018)	(2009–2014)	(2009–2014)	(2015–2018)	(2015–2018)
	(1)	(2)	(3)	(4)	(5)	(6)	(7)	(8)	(9)	(10)	(11)	(12)
Centrality × Regulation		.0003		.0011		.0002		−.0017^***^		−.0019^***^		−.0019^***^
		(.5817)		(1.0708)		(.1599)		(−2.6265)		(−2.5918)		(−2.9657)
Centrality		−.0038		−.0062		−.0035		.0139^**^		.0148^**^		.0182^***^
		(−.7849)		(−.8326)		(−.4042)		(2.3696)		(2.2426)		(3.2068)
Regulation	−.0023	−.0042	−.0068^**^	−.0128^***^	−.0005	−.0012	.0018	.0122^***^	.0037^*^	.0147^***^	−.0033	.0098^**^
	(−.7798)	(−1.4012)	(−2.4807)	(−4.9043)	(−.1197)	(−.1833)	(.7923)	(3.1373)	(1.6778)	(3.7230)	(−.6968)	(1.9810)
Constant	.1720^*^	.1804^**^	−.0595	.0159	.3377^***^	.3290^***^	−.0947	−.1821^***^	−.1206^*^	−.2191^***^	.0103	−.0892
	(1.7799)	(1.9696)	(−.7003)	(.1427)	(3.5679)	(3.8011)	(−1.5312)	(−2.6454)	(−1.9202)	(−3.1814)	(.0790)	(−.7921)
Control variables	Yes	Yes	Yes	Yes	Yes	Yes	Yes	Yes	Yes	Yes	Yes	Yes
Year	Yes	Yes	Yes	Yes	Yes	Yes	Yes	Yes	Yes	Yes	Yes	Yes
Industry	Yes	Yes	Yes	Yes	Yes	Yes	Yes	Yes	Yes	Yes	Yes	Yes
*N*	1,989	1,989	454	454	1,535	1,535	3,643	3,643	2,561	2,561	1,082	1,082
*R* ^2^	.1806	.1814	.2031	.2119	.1647	1674	.1637	.1662	.1499	.1537	.1856	.1893

* *Indicates significance at the levels of 10%*;

** *Indicates significance at the levels of 5%*;

*** *Indicate significance at the levels of 1%*.

### Heterogeneity Test of Peer CSR Practices

In the hypothesis development, another path for the relationship between board centrality and CSR decoupling is that firms learn the successful experience from their peers. Historical research based on network theory argues that firms with higher board network centrality gain an advantage in information from other firms, and learning from experience of others more quickly and efficiently (Larcker et al., 2013; Harjoto and Wang, 2020). For this explanation, we investigate whether board network centrality affects the firm’s learning on CSR practices from their peers.

We define variables CSP_peer and CSD_peer as the industry average of CSP or CSD in the last year, excluding the firm itself,^7^ and then design an interaction model as follows:


(4) $$\begin{array}{l} \begin{array}{l} {\mathrm{CSP}}_{i,t}/{\mathrm{CSD}}_{i,t}=\alpha_{0}+\beta_{1}{\mathrm{Centrality}}_{i,t-1}\\ \times \mathrm{CSP}\_{\mathrm{peer}}_{i,t-1}\left(\mathrm{CSD}\_{\mathrm{peer}}_{i,t-1}\right) \end{array}\\ \begin{array}{l} +\beta_{2}{\mathrm{Centrallity}}_{i,t-1}\\ +\beta_{3}\mathrm{CSP}\_{\mathrm{peer}}_{i,t-1}\left(\mathrm{CSD}\_{\mathrm{peer}}_{i,t-1}\right) \end{array}\\ \begin{array}{l} +\sum {\mathrm{Control variables}}_{i,t}\\ +\sum \mathrm{Year}\&\mathrm{Indu effects}+\varepsilon_{i,t} \end{array} \end{array}$$

where the dependent variables are CSP and CSD, respectively. CSP_peer (CSD_peer) represents the historical industry average level of CSP (CSD). Centrality×CSP_peer (CSD_peer) is the interaction term between the centrality variable and the industry average variable, which is our main interest. Control variables and year and industry effects are consistent with model (1).

Regression results are presented in Table 9. For brevity, we do not report the result for the whole period. Similar to section Heterogeneity Test of Regional Environment Regulation, first, we use variables CSP_peer and CSD_peer as the independent variables to regress, and investigate relationships between them and CSP/CSD. The regression results for the pre-adoption and post-adoption periods are reported in column (1), (3) and column (5), (7), respectively, indicating that lagged CSP_peer and CSD_peer have a significant positive effect on current CSP and CSD, which are consistent with the results of previous studies (Yang et al., 2017). Moreover, we use model (4) for regression. As shown in columns (2), (4), (6), and (8), the coefficients on the interaction terms Centrality×CSP_peer (CSD_peer) are both positive and more significant in the post-adoption period. Consisting with our theoretical expectation, board centrality improves firms’ learning of CSR practices from their peers, and this improvement effect is more pronounced in the period of the supervision strengthening.

**Table 9:** Heterogeneity test of peer CSR practices.

Variable	CSP	CSP	CSD	CSD	CSP	CSP	CSD	CSD
	Post = 0	Post = 0	Post = 0	Post = 0	Post = 1	Post = 1	Post = 1	Post = 1
	(2009–2014)	(2009–2014)	(2009–2014)	(2009–2014)	(2015–2018)	(2015–2018)	(2015–2018)	(2015–2018)
	(1)	(2)	(3)	(4)	(5)	(6)	(7)	(8)
Centrality×CSP_peer		.0524				.0635^*^		
		(1.3288)				(1.7167)		
CSP_peer	.6253^***^	.3233			.4861^***^	.0522		
	(4.6547)	(1.1724)			(3.2172)	(.1777)		
Centrality×CSD_peer				.0054				.0514^**^
				(.1583)				(2.1016)
CSD_peer			.4658^***^	.4371^**^			.3189^**^	−.0341
			(3.6339)	(2.2041)			(2.0769)	(−.2713)
Centrality		−.9077		−.0331		−1.0458^*^		−.3231^*^
		(−1.3645)		(−.1663)		(−1.6646)		(−1.7277)
CASH	−.1938	−.1784	.1021	.1022	.3424	.3068	−.0755	−.0926
	(−.6408)	(−.5618)	(.6508)	(.6418)	(.7401)	(.6646)	(−.7612)	(−.9527)
ROA	1.6132	1.0670	−2.1678	−2.1740	−.3544	.2786	−.4583	−.4251
	(.2866)	(.1883)	(−1.5614)	(−1.5605)	(−.0565)	(.0459)	(−.3431)	(−.3338)
BTM	−.3731	−.5492	−.2093	−.2102	−1.9649	−1.9269	−.2368	−.1720
	(−.2601)	(−.3891)	(−.5621)	(−.5533)	(−1.1665)	(−1.1600)	(−.6607)	(−.5008)
LEV	.6024	.6332	.1911	.1926	1.4681	1.2853	−.1830	−.2613
	(.2811)	(.2860)	(.3768)	(.3775)	(.7209)	(.6454)	(−.3112)	(−.4476)
SIZE	1.8334^***^	1.8391^***^	.3903^**^	.3902^**^	1.7589^***^	1.7815^***^	.6121^***^	.6098^***^
	(3.8000)	(3.8037)	(2.2420)	(2.2231)	(3.2972)	(3.2779)	(4.6979)	(4.6723)
AGE	−1.7267^***^	−1.7185^**^	−.4707^***^	−.4695^***^	−1.1756^*^	−1.1714^*^	−.6352^***^	−.6591^***^
	(−2.5892)	(−2.5601)	(−3.0513)	(−3.0702)	(−1.7005)	(−1.6900)	(−3.8831)	(−4.1198)
TOP1	.0357	.0354	.0083	.0084	.0027	−.0006	.0033	.0028
	(1.4392)	(1.4038)	(1.2482)	(1.2251)	(.0920)	(−.0210)	(.4820)	(.4051)
MSH	−2.6310	−2.6847	.5942	.5873	−1.0012	−1.0020	1.0200	.9815
	(−.4721)	(−.4796)	(.3785)	(.3755)	(−.4495)	(−.4411)	(.9685)	(.9697)
IB	−2.5635	−2.3022	.4344	.4482	−1.4543	−1.7068	.4268	.3029
	(−.3484)	(−.3127)	(.1761)	(.1834)	(−.2533)	(−.3000)	(.3158)	(.2304)
BSIZE	3.6262	3.9337	.7932	.8029	1.4048	1.3665	.5124	.3718
	(1.2838)	(1.3313)	(1.1640)	(1.1354)	(.7868)	(.6668)	(.9948)	(.7098)
DUAL	.5063	.5446	−.0512	−.0488	−.7233	−.7880	−.2978^**^	−.3080^***^
	(.6464)	(.7100)	(−.1749)	(−.1695)	(−1.3083)	(−1.4199)	(−2.5156)	(−2.6485)
Constant	−36.3616^***^	−32.2100^***^	−7.3216^***^	−7.1764^**^	−29.1723^***^	−21.6652^**^	−2.6817	.3390
	(−3.4830)	(−2.8693)	(−2.9910)	(−2.2899)	(−2.9235)	(−2.5258)	(−.9055)	(.1411)
Year	Yes	Yes	Yes	Yes	Yes	Yes	Yes	Yes
Industry	Yes	Yes	Yes	Yes	Yes	Yes	Yes	Yes
*N*	411	411	411	411	584	584	584	584
*R* ^2^	.4666	.4699	.4531	.4532	.3835	.3872	.4187	.4264

* *Indicates significance at the levels of 10%*;

** *Indicates significance at the levels of 5%*;

*** *Indicates significance at the levels of 1%*.

### Mechanism Test of Foreign Investor

Previous research suggests that foreign investors promote CSR engagement in emerging markets (Khan et al., 2013; Ali et al., 2017; Hao et al., 2018; Wang and Zhang, 2020). For example, Li et al. (2021) and McGuinness et al. (2017) find that firms invested by foreigners have better CSP in emerging markets. Hu et al. (2018) argue that foreign investors lead to a greater likelihood for CSR reporting in emerging markets. Thus, it is necessary to take into consideration the influence of foreign investors on the relationship between centrality and decoupling.

The influence of foreign investors on the relationship between centrality and CSR decoupling may be complex. The existing literature suggests that mature capital markets punish firms which adopt symbolic management in CSR practices (Marquis et al., 2016; García-Sánchez et al., 2020). However, in the market of investee firms, it is difficult for participants in the foreign market to supervise the CSR practices of investee firms due to the information limitations (Tashman et al., 2019). As a result, the investee firms easily respond to CSR pressure from overseas markets through symbolic practices (Jamali et al., 2015). The information strength brought by boards with high centrality helps their firms to better notice and to take advantage of the information disadvantage of the overseas participants, thus strengthening the use of symbolic strategy. In terms of under-decoupling, the influence of foreign investors is ambiguous, and the pressure from the overseas participants may stimulate under-decoupling firms with high board centrality to increase CSD and CSP at the same time, or only one of them, or even maintain the original strategy.

For the above argument, we design an interaction effect model as follows:


(5) $$\begin{array}{l} \begin{array}{l} \mathrm{Gap}\_{\mathrm{over}}_{i,t}/\mathrm{Gap}\_{\mathrm{under}}_{i,t}=\alpha_{0}+\beta_{1}{\mathrm{Centrality}}_{i,t-1}\\ \;\;\;\times {\mathrm{Foreign}}_{i,t-1} \end{array}\\ \begin{array}{l} \;\;\;+\beta_{2}{\mathrm{Centrallity}}_{i,t-1}\\ \;\;\;+\beta_{3}{\mathrm{Foreign}}_{i,t-1}\; \end{array}\\ \begin{array}{l} \;\;\;+\sum {\mathrm{Control variables}}_{i,t}\\ \;\;\;+\sum \mathrm{Year}\&\mathrm{Indu effects}+\varepsilon_{i,t} \end{array} \end{array}$$

where the dependent variables are CSP, CSD, Gap_over and Gap_under, respectively. Foreign is a dummy variable that equals 1 if there is a foreign investor in a firm; otherwise, it equals 0. Centrality×Foreign is the interaction term between the foreign investor variable and the centrality variable, which is our main interest. Control variables and year and industry effects are consistent with those of model (1).

Table 10 reports the results of model (5). Columns (1) and (2) show the coefficients on Centrality×Foreign in the pre-adoption period, which are not significant with the dependent variables of both decoupling. Columns (3) and (4) show the coefficients on Centrality×Foreign in the post-adoption period. The coefficient on Centrality×Foreign is significantly positive (*p* < .05) when the dependent variable is Gap_over. The above results indicate that, after regulation strengthening, foreign investors weaken the negative relationship between centrality and over-decoupling.^8^

**Table 10:** Mechanism effect of foreign investors.

Variable	Gap_over	Gap_under	Gap_over	Gap_under
	Post = 0	Post = 0	Post = 1	Post = 1
	(2009–2014)	(2009–2014)	(2015–2018)	(2015–2018)
	(1)	(2)	(3)	(4)
Centrality×Foreign	−.0027	.0001	.0035^**^	.0025
	(−.8293)	(.0414)	(2.1461)	(.6041)
Centrality	.0029^*^	−.0007	−.0035^***^	.0010
	(1.7942)	(−.6445)	(−2.7247)	(.4449)
Foreign	.0132	.0076	−.0247^*^	−.0155
	(.9687)	(.7464)	(−1.7887)	(−.5195)
CASH	.0025	.0018	.0021	.0063^**^
	(.3378)	(.4587)	(.7185)	(2.0199)
ROA	−.1659^***^	.1494^***^	−.0386	.0120
	(−2.7573)	(3.0925)	(−.7016)	(.3718)
BTM	−.0107	.0059	.0021	.0134
	(−.6955)	(.4761)	(.1228)	(.8383)
LEV	−.0164	.0397^**^	.0030	−.0025
	(−.5822)	(2.3070)	(.1847)	(−.1897)
SIZE	.0002	.0011	−.0119^***^	−.0029
	(.0241)	(.2035)	(−3.2608)	(−.7742)
AGE	.0013	−.0075	.0024	−.0029^***^
	(.1573)	(−1.6437)	(.5713)	(−3.0991)
TOP1	−.0001	.0001	−.0003	.0000
	(−.3963)	(.9619)	(−1.4911)	(.2675)
MSH	−.0012	−.0587^**^	−.0361^*^	−.0337
	(−.0221)	(−2.5500)	(−1.7465)	(−.5992)
IB	.0722	.0276	.0588	−.0186
	(1.4211)	(.6724)	(.9422)	(−.2253)
BSIZE	.0269	.0388^***^	.0092	.0152
	(1.2704)	(2.9798)	(.4596)	(1.0602)
DUAL	.0042	.0021	−.0034	−.0030
	(.2929)	(.2527)	(−.6799)	(−.3777)
Constant	−.0845	−.0799	.3410^***^	−.0081
	(−.9666)	(−1.1698)	(4.2373)	(−.0812)
Year	Yes	Yes	Yes	Yes
Industry	Yes	Yes	Yes	Yes
*N*	455	2,571	1,590	1,113
*R* ^2^	.1977	.1492	.1672	.1850

* *Indicates significance at the levels of 10%*;

** *Indicates significance at the levels of 5%*;

*** *Indicates significance at the levels of 1%*.

### Influence of Institutional Regulation Strengthening

The Chinese government began to implement mandatory CSR reporting policy since 2009. However, due to the lack of corresponding substantive guidance and supervision system, Chinese firms do not face high pressure on CSR legitimacy, and many firms only need to deal with it through symbolic strategies. For example, Kuo et al. (2012) find that up to 41% of 711 social reports released in 2010 (the 2nd year after the policy was adopted) provided little useful additional information, and only 17% of them reported quantification indicators on the firms’ CSR practices. Liao et al. (2018) find that the total ratio of CSR assurance for Chinese listed firms from 2008 to 2012 was only 4.04%, compared to the international level for sustainability reports from large firms assured by a third party of 40% (Kolk and Perego, 2010).

The new environmental law was adopted in 2015, which significantly enhanced the regulation strength (Zhang et al., 2017). The strengthening of CSR regulatory system causes more legitimacy pressure for firms. Especially when firms disclose more CSR information, they will attract more attention from stakeholders. Meanwhile, stricter legal basis enables the public to carry out rights protection litigation against firms and supervise enforcers. Based on 45 countries, Marquis et al. (2016) find that, in countries and regions with greater external supervision and institutional pressures, firms in the environmental sensitive industries disclose less selective CSR information. Based on polluting industries in the United States, Berrone et al. (2017) finds that the positive relationship between symbolic environmental practices and corporate legitimacy can be weakened when firms are strictly supervised by non-governmental organizations (NGOs). Yu et al. (2020) find that, based on transnational samples, higher scrutiny of independent directors and institutional investors can weaken the tendency of symbolic environmental practices. Therefore, after the strengthening of CSR regulatory system, firms with high board centrality have to be more careful about selective CSD and choose a more conservative reporting strategy, which weakens the positive relationship between centrality and over-decoupling, and makes centrality has a positive impact on under-decoupling.

For this concern, we employ a Differences-in-Differences design. The variable Post is defined as same as before, which equals 0 if the firm-years belong to 2009 to 2014, or 1 during 2015 to 2018. Specifically, the following estimation model was used:


(6) $$\begin{array}{l} \begin{array}{l} \mathrm{Gap}\_{\mathrm{over}}_{i,t}/\mathrm{Gap}\_{\mathrm{under}}_{i,t}=\alpha_{0}+\beta_{1}{\mathrm{Centrality}}_{i,t-1}\\ \;\;\times {\mathrm{Post}}_{i,t} \end{array}\\ \;\;+\beta_{2}{\mathrm{Centrallity}}_{i,t-1}\left(+\beta_{3}{\mathrm{Post}}_{i,t}\right)\\ \begin{array}{l} \;\;+\sum {\mathrm{Control variables}}_{i,t}\\ \;\;+\sum \mathrm{Year}\&\mathrm{Indu effects}+\varepsilon_{i,t} \end{array} \end{array}$$

where the dependent variables Gap_over/Gap_under represent over-decoupling and under-decoupling, respectively. Centrality × Post is an interaction term between the period variable Post and the centrality variable Centrality, which is our main interest. Control variables and year and industry effects are consistent with those of model (1). In addition, due to the multicollinearity between the period variable Post and the year effect variable, the period variable Post is removed in the regression when the year dummies are included (Chen et al., 2017).

Table 11 reports the results from estimating model (6). The dependent variables in columns (1) to (2) and (3) to (4) are the over-decoupling variable Gap_over and the under-decoupling variable Gap_under, respectively. Columns (1) and (3) involve the variable Post, the variable Centrality and their interaction term but not year dummies, because of the multicollinearity problem. Columns (2) and (4) involve all control variables and effects. Columns (1) and (2) show that the coefficients of Centrality × Post are −.0056 and −.0058, both significant at the 1% level, which suggests that the institutional regulation strengthening mitigates the positive relationship between centrality and over-decoupling. Columns (3) and (4) show that the coefficients of Centrality × Post are .0032 (*p* < .05) and .0020 (insignificantly). The results indicate that, after the institutional regulation strengthening, the relationship between centrality and under-decoupling tends to be negative in some kind. The above evidence suggests that the new environmental law brings significantly exogenous shock to the relationship between board centrality and CSR decoupling.

**Table 11:** DID approach.

Variable	Gap_over	Gap_over	Gap_under	Gap_under
	All	All	All	All
	(2009–2018)	(2009–2018)	(2009–2018)	(2009–2018)
	(1)	(2)	(3)	(4)
Centrality×Post	−.0056^***^	−.0058^***^	.0032^**^	.0020
	(−4.3926)	(−4.5889)	(2.4766)	(1.4344)
Centrality	.0011	.0028^**^	.0001	−.0007
	(1.1507)	(2.1437)	(.0756)	(−.6997)
Post	.0866^***^		−.0701^***^	
	(5.7804)		(−3.4085)	
CASH		.0014		.0025
		(.5499)		(.7920)
ROA		−.0792^*^		.0958^**^
		(−1.7157)		(2.1542)
BTM		−.0062		.0022
		(−.5832)		(.2905)
LEV		−.0063		.0227
		(−.4093)		(1.5438)
SIZE		−.0065		.0024
		(−1.3818)		(.6580)
AGE		.0002		−.0060
		(.0676)		(−1.6031)
TOP1		−.0003^*^		.0001
		(−1.8959)		(.6909)
MSH		−.0250		−.0579^**^
		(−1.3283)		(−2.4849)
IB		.0901^**^		.0049
		(1.9617)		(.1179)
BSIZE		.0126		.0322^***^
		(.9294)		(3.1388)
DUAL		−.0023		.0011
		(−.4665)		(.1608)
Constant	.0705^***^	.1261	.1461^***^	−.0885
	(4.3989)	(1.4161)	(5.7160)	(−1.4643)
Year	No	Yes	No	Yes
Industry	Yes	Yes	Yes	Yes
*N*	2,045	2,045	3,684	3,684
*R* ^2^	.1206	.1844	.0828	.1645

* *Indicates significance at the levels of 10%*;

** *Indicates significance at the levels of 5%*;

*** *Indicate significance at the levels of 1%*.

## Discussion

In this paper, we examine the impact of board network centrality on CSR decoupling. To test this relationship, we use a sample based to the Chinese capital market between 2009 and 2018. We reveal the complex role of the board network in CSR practices in China: (1) when the CSR institutional regulation is weak, board network centrality is positively related to over-decoupling but not related to under-decoupling and (2) when the regulation get strengthening, board network centrality is negatively (positively) related to over-decoupling (under-decoupling).

### Theoretical Contributions

First, our study extends the extant firm-related studies based on network theory. While the existing literature has demonstrated the relationship between board network centrality and CSP, the relationship between board network centrality and CSR decoupling is not examined further. Based on evidence from the Chinese market, we suggest that due to the information advantages of firms with high board centrality, it may enhance the symbolic management of CSR when CSR institutional regulation system is weak.

Second, our results support that the new environmental law plays an important role in strengthening CSR regulation in China. The law brings an incremental pressure on firms with high board centrality, making them to take more careful consideration about decision of CSD. Thus, the relationship between board centrality and over-decoupling (under-decoupling) turns to be negative (positive) after the adoption of the law.

### Managerial Contributions

Our results show that the influence of board network centrality on CSR practices is complex in the Chinese market. Firms with higher board centrality gain stronger advantages, including social capital and so on, and are more likely to have higher CSR over-decoupling when CSR institutional regulation system is weak, but the relationship switches to negative when the system is strong. This paper reminds CSD users such as SRIs that it is necessary to pay attention to the relationship between board network centrality and symbolic management when the CSR regulation is under developed.

### Limitation and Future Research

Firstly, limited by the availability of data, this paper only covers the cross-employment of directors and fails to include other types of networks, such as online networks when considering the calculation of board networks (Jing and Zhang, 2021). The solution to this problem needs the improvement of the availability of relevant data.

Moreover, our evidence is based only on the Chinese market, a special emerging market, which has a low level of legalization and public opinion supervision. These market characteristics may affect the CSR decoupling of Chinese firms to some extent. Therefore, whether the evidence of the relationship between board network centrality and CSR decoupling is established in other markets, especially mature capital markets, requires further investigation.

### Conclusion

This research examines the role of board network centrality in CSR decoupling based on network theory. Using the data of Chinese-listed firms between 2009 and 2018, it provides evidence that firms with higher board centrality may be more likely to implement symbolic strategies when the CSR regulation system is under developed, resulting in over-decoupling; but when the system get strengthening, the symbolic problem caused by higher centrality could be mitigated, meanwhile, higher pressure from regulation also makes firms with higher centrality increase under-decoupling. However, when regulatory pressure increases, they weaken the negative relationship between centrality and over-decoupling. We hope this paper could help SRIs and regulators better understand the complex impact of board networks on CSR practice. Furthermore, we suggest that the future research provide cross-country evidence about board network centrality and CSR decoupling.

## Data Availability Statement

The original contributions presented in the study are included in the article/supplementary material, and further inquiries can be directed to the corresponding author.

## Author Contributions

MZ and WZ contributed to the conception and design of the study, performed the statistical analysis, and wrote the first draft of the manuscript. XL, CY, and DD organized the database and wrote sections of the manuscript. All authors contributed to the article and approved the submitted version.

## Funding

This work was supported by National Natural Science Foundation of China (NSFC), Foundation number: 71902090 and China Postdoctoral Foundation, Foundation number: 2019M651843.

## Conflict of Interest

The authors declare that the research was conducted in the absence of any commercial or financial relationships that could be construed as a potential conflict of interest.

## Publisher’s Note

All claims expressed in this article are solely those of the authors and do not necessarily represent those of their affiliated organizations, or those of the publisher, the editors and the reviewers. Any product that may be evaluated in this article, or claim that may be made by its manufacturer, is not guaranteed or endorsed by the publisher.

## Footnotes

^1^Generally, firms that employ more directors of higher centrality have higher board centrality.

^2^Although widely used by previous studies, the Hexun rating has serious problems in terms of their indicator design, for example, rating scores are heavily influenced by financial performance, the shareholder dimension score accounts for nearly 70% of the total score, and the environmental dimension score only accounted for 0.3% in 2017 (Zhong et al., 2019).

^3^Their theoretical upper limits are 45 and 15.

^4^The new environmental law took effect on 1 January 2015.

^5^We use Centrality in year *t* + 1 as instrumental variable, so some firm-years with missing data are excluded in the regression, resulting in the decrease of sample size decreases in Table 6.

^6^In the China Environmental Almanac, the data of environmental penalty were only updated to 2015, so the values after 2015 are replaced by those of 2015.

^7^Since the peer learning effect can only exist in industries with a certain number of firms, we exclude sample industries with less than 10 firms.

^8^As shown in column (9) of Table 3, the coefficient on Centrality is −0.0026 and significant at 5% level.
